# The Rat—A Sanitary Menace and an Economic Burden

**Published:** 1913-09

**Authors:** R. H. Creel

**Affiliations:** Passed Assistant Surgeon, United States Public Health Service


### Abstracts and Selections.

### The Rat—A Sanitary Menace and an Economic Burden.

BY R. H. CREEL, PASSED ASSISTANT SURGEON, UNITED STATES PUBLIC HEALTH SERVICE.

Of all the parasites that have their being in and around the habitation of man the rat has less to justify its existence than any other. As devoid of any redeeming traits as the fly, which has been the subject of a nation-wide sanitary crusade, the rat is a greater pest because of its depredations and its possibilities for harm in the transmission and perpetuation of bubonic plague in a community. The latter consideration is of more serious import in seaport towns wherever they may be and in those localities where plague has once appeared, but with the world-wide march of bubonic plague in no city should its advent be considered as improbable.

Squirrels to the westward of the Rocky Mountains and the marmot in Asia are subject to the disease in a more or less chronic form, but these animals, on account of their infrequent contact with man, are a menace not so much in transmitting the disease to man as they are in being the source of a continued reintroduction of the disease among the neighboring rat population. It is, therefore, evident that the slogan "No rats,, no plague" is very expressive of fact.

A brief review of the rôle this animal plays in transmitting disease and in damaging and destroying property will easily convict it of being a most undesirable denizen.

No discussion of the part taken by the rat in spreading plague will be attempted, except to say that plague is, primarily and essentially, a disease of rodents, chiefly the different species of rat, and that it is conveyed to human beings from plague-infected rats through the agency of the fleas which infest the sick animal.

When plague has once gained a foothold in a country, the cost of stamping out the infection will be manifold the expense attendant upon the eradication of any other epidemic disease. The toll of human life may vary according to local conditions, but always the commercial prejudice against a plague-infected port and the expenditure for eliminative measures will result in heavy financial drain.

Turning from the aspect of a sanitary menace to an ever-present and continued commercial drain, the following is of interest. To assign any accurately fixed sum to the amount of injury done by rats in the United States is impossible, but, estimating the loss at a rational minimum amount, the sum is astounding. The calculation embraces two factors, namely, the rodent census and the average amount of damage done by one rat. Both of these factors can be determined within reasonable limitation.

For antiplague work in the United States and its insular possessions, the Philippines, Hawaii, and Porto Rico, there has been spent in recent years by the Federal Government, through the United States Public Health Service, and by the different local government forces, a vast sum. The loss to commercial interests in all these places, due to interference of shipping facilities and sanitary restrictions by other countries, has made the sum actually spent for plague work seem but a "drop in the bucket."

The scope of this article will not permit of an extended discussion of the sanitary aspect of plague, but it may be stated that the disease is endemic on every continent in the world and in practically all countries, excepting, possibly, those of continental Europe. In our own country any laxity of sanitary surveillance of the endemic centers on the Pacific coast would result in the broadcast spread of the disease. The same will apply to all endemic centers. It is a question of eternal vigilance.

By means of trapping percentages covering a period of one year it was determined that the rodent population in San Francisco was slightly in excess of the human population. In Porto Rico, where the same method of computation was employed, the proportion of rat and human inhabitants in cities was about equal.

In the rural districts of the United States the number of rats on any farm or plantation will easily average three or four times the number of people on the estate, and in the grain or cane producing areas the proportion will be multifold.

In cane-producing tropical and semi-tropical countries, such as Porto Rico, all the West Indies, the Hawaiian Islands, and the Philippines, where the roof rat and the field rat predominate, the rat population is incredibly large. On one cane plantation in Porto Rico where there were less than 500 people, within six months there were killed 25,000 rodents.

It is therefore evident that an estimate of the rodent population of the United States as equal to the human census would be well below the probable number. In our insular dependencies—Porto Rico, Hawaii, and the Philippines—where the cane fields are especially overrun with rats, the rodent population is undoubtedly several times the human population.

This estimate of one rat per human being for the continental United States coincides with that made for Great Britain and Ireland by the Incorporated Society for the Destruction of Vermin, and also with authoritative figures for Denmark, France and Germany.

The annual upkeep per rodent was computed by the same authorities as $1.80 in Great Britain, $1.20 in Denmark, and $1 in France. Judging from the large number of complaints made by American farmers in writing to agricultural journals, the depredations of rats in the country will exceed the estimate made in Great Britain. One-half cent per day would be a conservative estimate, however. The same figure can safely be placed on the damage caused by the city rat.

The list of articles damaged by rats is too long to enumerate in detail, but in general the following can be mentioned: All kinds of grain, before and after harvest; eggs and poultry, especially small chicks; wild birds, their eggs and young; fruits and vegetables, both while growing and when stored; flowers, bulbs, and shrubbery; all kinds of staples in bags or boxes; and all food products in pantries, groceries, meat markets, bakeries, stables, and general markets.

Lantz, in the Public Health Bulletin No. 30, "The Rat and Its Relation to Public Health," cites the following specific cases of rat depredation. Presumably they were selected at random:

"An Iowa farmer writing to an agricultural journal reported that rats had destroyed in one winter about 500 bushels of corn of a total of 2,000 bushels stored in cribs. Another farmer reported that rats had robbed him of an entire summer's hatching of three or four hundred chicks, and still another one attributed his loss in grain and poultry for one season due to rats as sufficient to pay his taxes for three years."

Lantz further quotes a Washington merchant to the effect that rats gnawed a hole in a tub containing 100 dozen eggs and within a period of two weeks carried away 71 dozen without leaving either shell or stain.

The writer once observed in San Francisco a shop dedicated to the sale of manicure supplies that was so rat infected that the proprietor had to move. The shop adjoined a bakery, and the depredations of the rats were so great that they actually entered a glass display case and gnawed the chamois skin on nail polishers. The reports of experimental stations in Guam, Hawaii, and Porto Rico lay special stress on the depredations of rats in the cane fields. Mr. R. L. Van Dine, of the Porto Rico experimental station, places the annual loss to cane growers in the island at $75,000, and states the loss is due not only to the cane actually destroyed, but also to the fermentation set up in the cane juices in the stalks that have been gnawed upon, which reduces the purity and sucrose content. This loss to Porto Rico planters was based upon the estimate that only one-half of 1 per cent of stalks were attacked by rats, but in reply to inquiries sent out by Van Dine the estimate made by different planters varied from 1 to 4 per cent of stalks attacked by rodents.

Because the rat is an animal of nocturnal habits, its depredations often pass unnoticed or are ascribed to other sources. Computing the upkeep of the rat as one-half cent per day, and estimating one rat to each person, the sum of $167,000,000 annually is lost to the country by the depredations of this pest.

A ratless country seems almost Utopian, but much can be accomplished in preventing this unnecessary loss and in safeguarding the country from any possible plague invasion, by a concerted and well-sustained nation-wide crusade against the rat similar to the "swat the fly campaign." No sporadic or individual effort will suffice.

The extermination of rats is not nearly so easy as fly destruction. An adult rat will on the average produce young six times yearly and from six to twelve in each litter. There have been known cases where a full-grown female littered twelve times in one year. A rat can reproduce when three months old. This remarkable fecundity, together with the instinctive secretive habits of the rat, which being an animal of noctural habits lies hidden during the day, and is active at night, while his human foe is asleep, readily accounts for the large rat population in any locality and emphasizes the difficulty of rat destruction.

Rats can be destroyed by trapping, by poisoning, and by using natural enemies, as certain breeds of cats and dogs. To insure success to these measures it will be necessary to curtail the rat's food supply by properly disposing of garbage and table refuse and by preventing rats from gaining access to such food as is contained in pantries, groceries, markets, stables, etc. The municipal government will have to assist the efforts of citizens along this line by creating and enforcing suitable rat-proofing laws.

To merely keep premises clean and free of rubbish will be of but little benefit, as rodents generally, even when abundant rubbish is available, prefer more secure covert, as that beneath floors, and within double walls and ceilings. So along with other measures for the destruction of rats all buildings, chicken yards, garbage receptacles, sidewalks, and planked areas must be built or repaired to prevent rat harborage.

The rat-proofing of buildings is generally secured either by elevation of the structure, with the underpinning open and free, or by marginal rat-proof walls of concrete, or stone or brick laid in cement mortar, sunk two feet into the ground, fitting flush the floor above. The wall must fit tightly to the flooring and not merely extend to the joists or supporting timbers, as this would result in open spaces for the entrance of rodents. Groceries, stables, warehouses, markets, and food depots in general are best rat-proofed by having a concrete floor in addition to the walls. In these structures, untenanted as they are at night-time, rats might well enter by a doorway or window carelessly left open or be introduced concealed in merchandise, and gnawing through plank flooring obtain a well-protected hiding and breeding place.

In addition to concrete floors and walls these food depots must have tight-fitting doors, and all windows and openings should be properly screened. A 12-gauge wire is preferable on account of its strength and durability, and the mesh should not be larger than one-half inch.

Rat-proofing by elevation is chiefly applicable to small and medium size frame dwellings. The intent is to have sufficient elevation, about two feet, so that the ground area beneath will be as exposed and free from covert as unbuilt upon land. Marginal rat-proofing will suffice in more pretentious dwellings where sufficient care can be exercised to prevent rats from gnawing through the plank floors.

Chicken pens can be protected by concrete walls at the periphery, sunk into the ground two feet or more with one-half-inch mesh wire netting, covering sides and top. Garbage cans should be of serviceable metal with properly fitting tops.

Plank sidewalks and plank coverings for yards should be avoided. Cinders or concrete are preferable for this purpose. The latter should have marginal protection to prevent rats from burrowing beneath it.

Double walls with a dead space between them should be avoided or if used, they should be rat-proofed at top and bottom with heavy wooden timbers, 4x4 joists, or by a concrete fill. Attics should be well opened and kept free from dunnage or other refuge for rats.

These precautions gainst rat harborage and for the protection of food supplies, in connection with careful trapping and poisoning will be attended with considerable success toward the destruction of rats.

As to trapping and poisoning, it may be stated that the efficacy of these measures will depend not so much on the kind of poison or on the pattern of the trap, or the bait, as upon the method of placing the poison and traps. The larger the wire-cage trap the better the results. It goes without saying that both the snap traps and the cage traps should be substantially made, and the latter should have wires well reënforced.

There are several important points about placing traps. They should be placed wherever rats have been accustomed to frequent for feeding purposes. Traps should be more or less concealed, the small snap traps by scattering dust, flour, or cornmeal on and about them, and the cage traps by pieces of sacking, straw, or rubbish, leaving only the opening free. The prerequisite of successful trapping is that no food other than the bait should be available to the foraging rodent. Other things being equal, highly savory articles, such as cheese and toasted bacon, will more quickly attract rodents than will food without odor, but the idea that a rat can be enticed into a trap by the employment of bait more appetizing to him than the surrounding food supply is fallacious. To the rat, food supply is a question of availability, not preference. A number of specific cases have impressed this upon the writer. In one instance where a bakery was overrun with rats, a most experienced trapper set traps in and around the place for two or three weeks without catching a single rodent. This barren result continued notwithstanding the rotation of bait. Cheese, bacon, meat, vegetables, flour, nuts, and every known kind of bait in turn was used without avail. The rodents played and cavorted about the traps but never entered. Finally the bakery was moved and the building closed preparatory to rat-proofing. Three or four days after the removal of the stock, when all loose flour and food had been consumed by the rats, the trapper caught over thirty rats in one morning and in four days the place yielded a bag of some eighty rodents.

Traps or poison placed in the neighborhood of an overflowing garbage pail, in a pantry with open bins and exposed food, or in groceries and warehouses having foodstuffs spilled over the floor, will only result in wasted endeavor.

Trapping is preferable to poisoning, for the reason that the results are accurately known, whereas in poisoning, the result is always a matter of conjecture. Both methods should be employed, however. For the individual householder any of the poisons obtainable in open market and which have arsenic, phosphorus, or strychnine as the active ingredient, will be effective if properly used.—*Reprint from the Public Health Reports,* Vol. 28, No. 27, July 4, 1913.

